# Antioxidant-rich dietary intervention for improving asthma control in pregnancies complicated by asthma: study protocol for a randomized controlled trial

**DOI:** 10.1186/1745-6215-15-108

**Published:** 2014-04-04

**Authors:** Jessica A Grieger, Lisa G Wood, Vicki L Clifton

**Affiliations:** 1 Robinson Institute, School of Paediatrics and Reproductive Health, Adelaide University, Lyell McEwin Hospital, Haydown Road, Elizabeth Vale, SA 5112, Australia; 2 Hunter Medical Research Institute, School of Biomedical Science and Pharmacy, University of Newcastle, Kookaburra Circuit, New Lambton Heights, NSW 2305, Australia

**Keywords:** Antioxidants, Pregnancy, Asthma, Asthma control, Diet

## Abstract

**Background:**

Asthma is the most prevalent chronic disease to complicate pregnancies worldwide, affecting around 12% of pregnant women in Australia. Oxidative stress and inflammation manifest during pregnancy; however asthma in pregnancies further intensifies oxidative stress. Consumption of antioxidant-rich foods has been shown to be beneficial for asthma control in non-pregnant asthmatic adults. It has not been investigated whether antioxidant-rich foods can improve the elevated oxidative stress that occurs with asthma in pregnancy, thereby improving asthma control. The primary aim of this study is to determine whether increased consumption of antioxidant-rich foods for 12 weeks will improve maternal asthma control, compared to standard dietary intake during pregnancy.

**Methods/design:**

A 12 week, parallel randomized controlled trial will be conducted. One hundred and sixty eight pregnant women with mild, moderate, or severe asthma, currently using inhaled corticosteroids, and with poor diet quality, will be recruited at approximately12 weeks gestation. Following a 4 week run-in period, women will be randomized to either a 12 week antioxidant intervention (increased consumption of antioxidant-rich foods (≥5 servings/day vegetables, ≥2 servings/day fruit, ≥8 ½ servings/day grains (mostly wholegrains), 3–4 serving/week lean meat) or standard pregnancy care. The primary outcome is asthma control score (decrease of 0.5, the minimally clinically significant change). Secondary outcomes include plasma antioxidants, markers of oxidative stress, and time to, and number of, exacerbations. With two-tailed t-tests at 80% power, a sample size of 52 completions per group is required. Allowing for a 78% retention including a 20% removal of women from the analysis due to non-compliance, we will recruit 168 women.

**Discussion:**

It is expected that this 12 week study will improve asthma control. This is significant because asthma is the most prevalent condition to complicate pregnancies and contributes to poor maternal, neonatal and infant health outcomes. Our research will provide the first evidence to show that, in pregnancy, consumption of antioxidant-rich foods is a key modifier of clinical asthma status. This research is crucial for contributing to the evidence base to inform future guidelines given existing clinical and research gaps.

**Trial registration:**

ACTRN12613000301763

## Background

Asthma is a chronic and complex inflammatory disease of the airways with symptoms including excess mucus production, wheeze, dyspnea, cough, fatigue, anxiety, tachycardia, and chest tightness. Oxidative stress plays a role in asthma etiology due to activation of various inflammatory cells of the respiratory tract such as neutrophils, eosinophils, mast cells and lymphocytes [1]. The continuous exposure of the respiratory tract to environmental oxidants and airway inflammatory cell-generated reactive oxygen species creates a high level of oxidative stress in the lung [2,3].

The lungs have endogenous antioxidant mechanisms to combat the damaging effects of reactive oxygen species; however, levels of antioxidants in the lungs as well as in circulation are reduced in asthmatic patients [4-6]. Wood *et al*. established for the first time that in adults with asthma, α-tocopherol levels were reduced in subjects with severe disease compared with those with a mild-to-moderate asthma pattern; α-tocopherol levels were also reduced in those with airway hyper-responsiveness; and in patients with stable, but poorly controlled asthma, antioxidant potential was lower compared with those with controlled or partly controlled asthma [7]. Other human mechanistic studies have shown that the airway epithelium in asthmatic adults is more susceptible to oxidants than in non-asthmatic adults [8], and Zalewski *et al*. identified lower labile sputum zinc levels that were associated with increased frequency of wheeze, asthma severity and reduced lung function [9]. In an intervention study, asthmatic subjects who withdrew antioxidant-rich foods from their diet for 10 days, including limiting fruit (≤1 serving/d) and vegetable (≤2 servings/d) intake (n = 22), had reduced plasma carotenoids, increased sputum neutrophils and poorer lung function and asthma control [10]. Comparatively, increasing dietary intakes of carotenoids, through increased consumption of fruit (≥2 servings/d) and vegetables (≥5 servings/d) for 14 weeks was shown to positively affect clinical asthma outcomes in adults with asthma, by reducing the risk of asthma exacerbation [11]. Importantly, improvements in asthma control only occurred after increased intake of fruit and vegetables, whereas no improvements in asthma outcomes were identified following 14 weeks of lycopene supplementation [10]. Other studies assessing single antioxidant supplementation in asthmatic adults do not provide support for improving asthma outcomes [12-14]. Therefore, dietary modification through whole foods appears a valuable strategy for improving outcomes associated with asthma.

Asthma is the most prevalent chronic disease to complicate pregnancies worldwide, with prevalence between 8 and 13% [15-17]. In Australia, about 12% of pregnant women have asthma, and 35% of these women report that their asthma worsens during pregnancy [16]. Clifton *et al*. identified that asthma in pregnancy increases risk of low birth weight, small-for-gestational-age (SGA) babies, preterm delivery and preeclampsia [18]. Asthma exacerbation is frequent, even in mild asthma; this has been linked to increased risk of low birth weight [19].

Oxidative stress is manifested at the maternal-fetal interface and contributes to normal placental development. The progression of pregnancy increases the activation of several maternal oxidative stress and inflammatory pathways [20] and compared to the non-pregnant state, markers of oxidative stress are higher [21]. In addition, the placenta is believed to be a major source of free radicals and lipid peroxide production [21], leading to systemic oxidative stress during gestation and delivery. Generally, pregnant women cope with these physiologic changes which are controlled by an increase in antioxidant enzyme activity [22,23]. Asthma during pregnancy has been associated with increased markers of oxidative stress, independent of asthma severity or treatment [24] and may promote the worsening of asthma through systemic inflammation and local production of chemokines from airway cells [25].

Maternal nutrition has the potential to influence fetal growth, as well as immune and airway development. However, there has been a shift from higher consumption of traditional plant-based foods to diets higher in processed foods [26]. This increased intake of processed foods and higher intake of fat, specifically saturated fat, and sugar, parallels the reduced intake of fruits and vegetables that contain antioxidants [27,28]. Poor diet quality during pregnancy has been associated with adverse fetal and childhood outcomes: a low intake of fruit and vegetables increased the odds of childhood wheeze [29] and a high intake of butter and butter spreads increases risk of childhood rhinitis [30]. Comparatively, dietary patterns that include higher intakes of fruit and vegetables are associated with reduced odds for growth restriction [31] and SGA babies [32,33]. Supplementation studies assessing multiple micronutrients [34], vitamin D [35], vitamins B or C [36], vitamins E or C [37,38] or vitamin A [39] did not improve maternal or perinatal outcomes (for example, preterm birth, low birth weight, perinatal morbidity, or preeclampsia). Other studies have suggested vitamin E supplementation might adversely affect maternal health [40] and an antioxidant supplement might increase risk of preterm delivery [41]. Altering food intake patterns towards an antioxidant-rich diet, which is also fiber-rich and low in fat, might be protective in asthma.

There are few studies of dietary intake in pregnant women with asthma. One study found that pregnant asthmatic women with low circulating concentrations of individual antioxidants had poorer fetal growth outcomes such as head circumference and birth weight, but no associations were found for non-asthmatic women [42]. These data suggest a link between poor perinatal outcomes and maternal antioxidant status. Interventions aimed at improving asthma control and that might subsequently impact perinatal outcomes deserve investigation.

Improving antioxidant status and relieving the oxidative stress that occurs with pregnancy is likely to have a beneficial effect. Nutritional strategies that can lower maternal oxidative stress may premise for preventing the asthma burden in pregnancy. Given the often poor dietary intakes during pregnancy, the increasing rate of pregnancies complicated by asthma, and the prevalence of adverse maternal and fetal outcomes among pregnant women with asthma, it is timely to intervene in this population with antioxidant-rich foods. Pregnancy is considered a key time for dietary modification, as several adaptations take place in which additional nutrients are needed to support development. Currently, it has not been investigated whether increasing consumption of antioxidant-rich foods, in pregnant women with asthma, will demonstrate improvements in asthma control. The potential for diets containing antioxidants to increase maternal antioxidant defence provides a valuable strategy that could subsequently reduce oxidative stress and improve asthma control. Improved diet in combination with asthma management and treatment will improve asthma control and therefore improve fetal outcomes.

The aims of the proposed research are to determine, in pregnant women with asthma, whether 12 weeks consumption of antioxidant-rich foods (≥5 servings/d vegetables, ≥2 servings/d fruit, ≥8½ servings/d grains (mostly wholegrains), 3 to 4 servings/week lean meat)) which meets nutrient requirements during pregnancy will: 1) improve asthma control; 2) improve antioxidant concentrations, and reduce markers of oxidative stress and inflammation, and 3) reduce the risk of asthma exacerbation, compared to standard dietary care during pregnancy.

## Methods/design

### Study design

We will conduct a 12-week, parallel randomized controlled trial (RCT). Pregnant women with asthma will be randomly allocated to either 1) an antioxidant-rich diet (that is, increased consumption of foods containing antioxidants; ≥5 servings/d vegetables, ≥2 servings/d fruit, ≥8½ servings/d grains (mostly wholegrains), 3 to 4 servings/week lean meat, or 2) standard care during pregnancy.

### Ethics and trial registration

Ethics approval for this study was granted by the Human Research Ethics Committee (TQEH/LMH/MH) (project: HREC/13/TQEHLMH/200). The trial was registered on the Australia and New Zealand Clinical Trials registry (ANZCTR) on 19 March 2013 (ANZCTR Number: ACTRN12613000301763).

### Participants

Pregnant women will be recruited from Lyell McEwin Hospital (LMH), Adelaide, Australia. The LMH is situated in a region of lower socioeconomic status, as determined by the South Australian Health Atlas. Inclusion criteria are as follows: pregnant women with mild, moderate, or severe asthma and currently using inhaled corticosteroids (ICS) (see Assessment of asthma below); >18 years of age, and poor diet quality (<3 servings/d fruit and vegetable). Exclusion criteria are as follows: recent (past month) respiratory tract infection; intermittent asthma; current smoker; use of antioxidant supplements, or previous pregnancy complications including growth restriction, stillbirth or preterm delivery.

### Protocol

**Figure 1:**
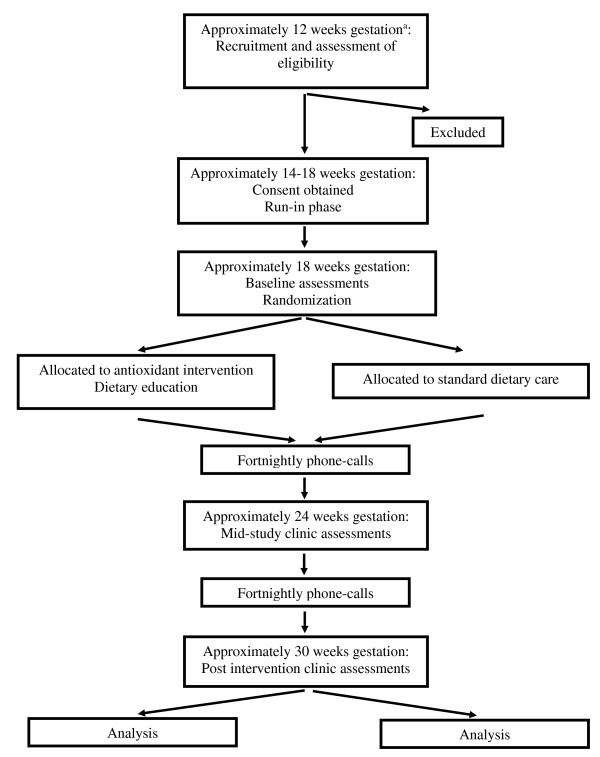
**Flow diagram of the progress through the phases of the randomized controlled trial. **^a^Women attending their first antenatal visit at 10 to 14 weeks gestation will be recruited. The run-in phase and intervention will start 2 weeks and 4 weeks following recruitment and consent.

At approximately 12 weeks gestation (range 10 to 14 weeks), asthmatic women will be recruited to participate (Figure 1) in the 12-week intervention study, with an initial 4-week run-in period (Table 1). At this initial visit, women will have the study procedures explained and have their asthma assessed (described below). A telephone call to determine consent will be made at approximately 13 weeks gestation in which a date to start the subsequent run-in period will be made. Signed consent forms will be obtained at the 14 weeks gestation visit. At this visit, the run-in period will commence where the women will be provided individual asthma management and education, complete a questionnaire on how/if their food intake has changed since learning they were pregnant, and will complete a food frequency questionnaire (FFQ). At 18 weeks gestation (that is, the baseline visit), women will be randomized into a control or intervention group (Table 1). At this baseline visit, weight and blood pressure will be measured, a fetal scan performed, questionnaires on asthma control, physical activity, and smoking will be completed, a blood sample taken, and dietary information collected. The same measurements will occur at 24 and 30 weeks gestation for all women. Phone calls will be made at 20, 22, 26 and 28 weeks gestation for information on asthma control and dietary intakes, and women in the intervention group will receive additional dietary counseling and support regarding the consumption of antioxidant-rich foods. At 30 weeks gestation, both groups will come back to the clinic for their final visit (Table 1). At delivery, birth outcomes will be assessed.

**Table 1:** Study endpoints and procedures for the intervention and control groups

**Week of gestation**	**12**	**14 (n = 168)**	**16**	**18**	**20**	**22**	**24**	**26**	**28**	**30 (n = 104)**
**Endpoint**	**Recruit**	**Run-in**		**Randomization and intervention**						**End**
**Clinic**										
Height		X								
Weight		X		X			X			X
Blood pressure		X		X			X			X
Fetal scan				X			X			X
FFQ		X								
24-hr recall		X		X			X			X
Blood		X		X			X			X
AME		X		X						
ACQ6	X	X		X			X			X
F_E_NO		X		X			X			X
Questionnaires^a^				X			X			X
Food group count	X			X			X			X
**Telephone**										
ACQ6			X		X	X		X	X	
Food group count			X		X	X		X	X	
24-hr recall			X		X	X		X	X	

FFQ, food frequency questionnaire; AME, asthma management and education; ACQ6, asthma-control questionnaire 6; F_E_NO, fraction of exhaled nitric oxide. ^a^Questionnaires include: eating habits subscale, common-cold questionnaire, passive-smoking questionnaire and physical-activity questionnaire.

The control group will receive only the standard-care LMH pregnancy booklet, *Healthy Eating During Pregnancy*. The booklet contains the recommended number of servings of each food group to be consumed in pregnancy, according to the 2013 Australian Dietary Guidelines (http://www.nhmrc.gov.au/_files_nhmrc/publications/attachments/n55a_australian_dietary_guidelines_summary_131014.pdf), as well as further information on key nutrients during pregnancy, supplements, weight gain during pregnancy, and guidance on, for example, alcohol, caffeine and water intake, and listeria, as referenced from SA Health; RANZCOG 2009; Noarlunga Health Services; and Food Standards, Australia and New Zealand. No additional dietary education will be provided to the control group.

The intervention group will receive the same booklet; however, the group will be further counseled on types of antioxidant-rich foods to purchase and consume such as fruits (for increasing intake of carotenoids, vitamins A and C), vegetables (carotenoids, vitamins A and C), wholegrains (vitamin E, selenium) and lean meat (zinc, iron, long chain omega 3 fatty acids) over the 12-week study. Examples of types of foods to consume will also be provided: for example, choosing spinach leaves instead of lettuce, and sweet potato instead of white potato. Women will be provided with a list of foods that are high in antioxidants, and will be asked to identify which types of these foods they are most likely to purchase and consume over the 12 weeks. Women in this group will also be given meal/snack suggestions to assist compliance. Previous pilot work from the authors has identified, in a dietary pattern analysis, that a high fat/sugar/takeaway pattern was evident in this population of pregnant women with high consumption of takeaway foods, crisps, refined grains and cakes and limited consumption of fruits and vegetables. Therefore, a shopping voucher of $30 per week per intervention participant will be provided, which will contribute to the cost spent on fruits and vegetables, as well as wholegrains and lean meats.

### Assessment of asthma and asthma outcomes

Women attending their first booking visit at the LMH antenatal clinic will be identified with asthma by the attending midwife. To determine asthma, the midwife will ask, ‘Have you been told by a doctor that you have asthma?ʼ and ‘Have you used any asthma medications in the last year like Ventolin or a preventer?’ Interested asthmatic women will have the study explained with written and verbal information by the research midwife. At 14 weeks gestation, women will come back to the clinic to start the run-in phase in which they will have an asthma assessment determined by current asthma therapy and control, current asthma triggers and comorbidity and past history including frequency of oral corticosteroid use and previous hospital admissions for asthma. At this first visit, all women will undergo spirometry to assess lung function before and after salbutamol use. Measurements will be taken by a trained respiratory nurse. Blood will be drawn for a full blood count and total IgE to assess atopy.

### Asthma control

Asthma control will be assessed using the standard asthma control questionnaire 6 (ACQ6) [43] administered by the respiratory nurse. Controlled asthma will be defined as ACQ <0.75, partly controlled asthma will be defined as 0.75 to <1.5, and uncontrolled asthma will be defined as ACQ >1.5 [43]. Information on evidence of infections, cold or flu will be obtained using the common-cold questionnaire [44] at each visit. Asthma severity will be classified based on their current symptoms as mild, moderate or severe [45]. Exacerbations prior to the visit will be assessed by noting unscheduled doctors’ visits, increases in medication requirements, emergency department visits, or hospitalizations.

### Fractional exhaled nitric oxide (F_E_NO)

F_E_NO measures airway inflammation and asthma control (NIOX, Aerocline, Solan, Sweden). We will measure F_E_NO at a controlled flow rate of 50 mL/s according to the American Thoracic Society guidelines, and as reported in Murphy *et al*. [46].

### Asthma management and education(AME)

Standard management of asthma will be carried out by the respiratory nurse [47,48]. At the run-in phase, all asthmatic women will be provided information on how to manage their asthma, and be given asthma education and advice. This 30-minute session will include skills assessment (medication adherence, inhaler device technique); development of a written asthma action plan and advice on trigger avoidance, and education about asthma control. It is important that the women in the study receive this, as previous data obtained from LMH indicate that 16% of pregnant women have asthma and it is often poorly controlled, leading to high exacerbation rates. Women assessed as unstable and requiring medical review will be referred to a respiratory physician. Urgent medical review will be available for patients with an acute exacerbation. Women will be encouraged to see their family doctor if their asthma worsens and general practitioners (GPs) will receive information relating to the study so that they are aware of its purpose.

### Maternal and perinatal characteristics

Maternal height, weight, age, weight gain, blood pressure, parity and gravidity will be recorded. Socioeconomic status will be collected, including postcode, maternal education and occupation, marital status and paternal education and occupation (if available). Labor information will be collected and neonatal data will be collected at delivery, including gestational age at delivery, birth weight, length, head circumference, Apgar scores and any congenital abnormalities. Birth-weight centiles will be calculated using http://www.gestation.net. Fetal birth weight will be categorized as low birth weight (LBW: <2500 g); small for gestational age (SGA, <10th percentile for gestational age); intrauterine growth restriction (IUGR, <3rd percentile for gestational age); large for gestational age (LGA, >90th percentile for gestational age); and macrosomia (>4,000 g). Gestational age will be determined by date of the last menstrual period and 18-week ultrasound.

### Dietary measurements

At 14 weeks gestation (run-in phase) all women will firstly complete a questionnaire on pregnancy food intake. This questionnaire will capture information on how/if they have changed their diet since identifying they are pregnant. Weeks of gestation will be recorded for both the time they found out they were pregnant and current gestation. Questions on how they have changed their diet will be asked, including reference to each of the food groups in the *Australian Dietary Guidelines*, plus alcohol consumption, soft cheese and deli meat consumption (to assess risk of listeria), fish consumption, and intake of non-core foods, such as takeaway foods. Next, all women will complete the standard Cancer Council of Victoria’s Dietary Questionnaire for Epidemiological Studies (FFQ) to assess food intake 1 year prior to pregnancy. A 24-hr recall will be administered at the face-to-face visits and over the phone. This questionnaire asks for detailed information on meals and snacks consumed 24 hr prior to the clinic visit. Data will be entered into the Foodworks (Xyris, Brisbane) database. Throughout the 12-week study, all women will be required to complete a weekly count of the average number of servings of each food group consumed according to the *Australian Dietary Guidelines*. Eating habits will be measured using the eating habits subscale from the Project EAT Survey [49]. All items are scored on a scale of 1 to 5 and summed, a higher score indicating less desirable eating habits.

### Questionnaires

A questionnaire on passive smoking will be included, as passive smoking can play a role in asthma severity and antioxidant status. A passive-smoking questionnaire previously used by our colleagues [46] will be carried out at baseline and at the middle and end of the 12-week study. A validated, 10-minute self-administered physical activity questionnaire for pregnant women [50] will also be conducted and modified to cover the prior weeks of physical activity (Table 1). Significant differences in baseline passive smoking or physical activity variables will be controlled for in the analyses.

### Biochemistry

At each clinic visit, 20 mL of non-fasting, whole blood will be taken from the median cubital vein and collected into an ethylenediaminetetraacetic acid (EDTA)-coated tube. Plasma carotenoids, including lycopene, lutein, β-cryptoxanthin, α-carotene, and β-carotene will be analyzed using high-performance liquid chromatography [51]. Plasma inflammatory markers (IL-1, IL6, TNF-α) will be measured using standard ELISA assay procedures (Cayman Chemical, Ann Arbor, MI, USA). Total antioxidant potential will be measured using calorimetric assay (Sapphire Bioscience, Waterloo, NSW, Australia). Plasma zinc will be measured using atomic absorption spectrometry. Erythrocyte superoxide dismutase activity will be measured by colorimetric assay (Bioxytech; OXIS International, Portland, OR, USA). Total fatty acids in red blood cell membranes will be analyzed by gas chromatography [52].

### Achieving compliance

Compliance with the intervention is critical to the success of this project and will be achieved using behavior change strategies including self-monitoring, and regular phone calls for dietary counseling. The $30 shopping voucher will be used to assist compliance as this population typically has a low intake of antioxidant-rich foods. All participants will complete a food group diary and regular 24-hr dietary recalls, and the intervention group will have additional dietary education during the phone calls. We have previously achieved good compliance with this intervention using similar strategies [10,11]. To monitor compliance, we will measure blood carotenoids for the increased fruit and vegetable consumption. We will also collect the shopping voucher receipts and document amounts of foods purchased.

### Randomization

The randomization schedule will be created by an independent statistician using ralloc.ado version 3.6.1 in Stata version 11.1. Randomization will be stratified by pre-pregnancy asthma severity (based on the Global Initiative for Asthma guidelines) and body mass index.

### Sample size and recruitment

The primary outcome for this study is asthma control (ACQ6). In our previous study, we saw a change in asthma control of 0.4 (SD = 0.9) following 10 days on a low-antioxidant diet [10]. In this trial of longer duration we expect to see a clinically significant change in ACQ of 0.5. Using PS.exe (Power and sample size calculation, version 3.0.43) with two-tailed *t*-tests at 80% power, we have determined that a sample size of 52 completions per group is required. The previous trial achieved 78% retention; however, 20% were removed from the analysis due to non-compliance. Therefore, we will recruit 168 women so that 104 women (52 per group) will complete the study and be included in the analysis. There are 3,600 births per year at LMH and at least 12% of the women have asthma. From our previous cohort studies at the LMH, we have found that approximately 360 pregnant women have a complication of asthma. We will need to recruit a minimum 84 per year (including 20% low compliance + 22% drop out) to complete the study within 3 years. We believe capturing this number of women per year is feasible, given we only need to recruit 23% of the asthmatic women presenting to the Antenatal Clinic.

### Statistical analyses

All statistics will be performed using SPSS version 20.0 (SPSS, Inc., Chicago, IL, USA). Frequencies and descriptive data for the study population will be reported as mean (SD) or between groups as mean (± standard error of the mean). Prior to hypothesis testing, the distribution of the data will be examined for normality. Any skewed data will be natural log-transformed. Outcome comparisons will be made according to the treatment allocation at randomization on an intention-to-treat basis. Both adjusted and unadjusted analyses will be carried out. Continuous variables with repeated measurements (that is, ACQ6 score, blood results) will be analyzed using a generalized linear mixed model with a random intercept for individuals, to account for repeated measurements. Planned sub-analyses will be undertaken to assess the effects of asthma severity, asthma control, forced expiratory volume in 1 second (FEV_1_) and F_E_NO, on the primary and secondary outcomes. We will account for changes in ICS by assessing ICS intake as a cumulative dose over the study period. Differences between groups in time to exacerbation will be analyzed using Kaplan-Meier curves. All tests will be conducted two-sided, and a *P*-value <0.05 will be considered statistically significant.

## Discussion

Pregnant women have heightened inflammation and oxidative stress and this is further compounded by asthma. The lungs have endogenous antioxidant mechanisms to combat the damaging effects of reactive oxygen species; however, as levels of antioxidants in the lungs as well as in circulation are reduced in asthmatic patients [4-6], balancing the oxidant-antioxidant system may ameliorate oxidative stress in pregnancies complicated by asthma.

In non-pregnant asthmatic adults, epidemiological studies provide mixed evidence for the use of dietary antioxidant vitamins or antioxidant-rich foods to improve asthma. Current evidence from RCTs also suggests that supplementation with individual antioxidants is not beneficial in improving lung function or asthma control. Only one RCT has examined the effect of manipulating antioxidant intake, via the consumption of whole foods, on asthma outcomes and lung function in asthmatic adults [11]. Specifically, compared to a high fruit (≥2 servings/d) and vegetable (≥5 servings/d) diet for 14 weeks, the asthmatic subjects who consumed a low fruit (<1 serving/d) and vegetable (<2 servings/d) diet had a 2.26-fold increased risk of asthma exacerbation [11]. No studies have assessed antioxidant supplementation or dietary modification with antioxidant-rich foods in pregnant women with asthma. It is likely that these women would benefit most from such an intervention in order to improve asthma control and potentially improve birth outcomes.

The proposed study aims to determine whether consumption of an antioxidant-rich diet for 12 weeks, including fruits, vegetables, lean meat and wholegrains, will improve asthma control and improve antioxidant concentrations, reduce oxidative stress markers, and reduce the risk of asthma exacerbation, compared to standard dietary intake during pregnancy. The necessity of adherence to a diet rich in antioxidants in this study is crucial. However, we will use behavior-change strategies such as self-monitoring and regular phone calls for dietary counseling. This will ensure that all women have regular contact with the researchers to achieve adherence, as well as to ask any questions related to problems in achieving adherence. All women will complete a weekly food group diary to promote adherence and women in the intervention group will also have fortnightly phone call sessions with the nutritionist who will provide education on meal plans and antioxidant-rich food options. All of these strategies will be in place to ensure a successful intervention.

This 12-week study aims to improve asthma control, maternal plasma concentrations of antioxidants and decrease rates of asthma exacerbations. These outcomes are significant because asthma is the most prevalent condition to complicate pregnancies, and asthma in pregnancy leads to poor maternal, neonatal and infant health outcomes. Our research will provide the first evidence to show that, in pregnancy, dietary antioxidant consumption is a key modifier of clinical asthma status. Our research will provide the first step towards developing larger clinical trials through which increased antioxidant-rich foods will lead to a reduced risk of low birth weight, growth restriction, preterm delivery, and preeclampsia, via an improvement in asthma control; this will also potentially reduce the risk for atopic disease in childhood. These outcomes will provide further evidence to show that significantly increasing dietary antioxidant intakes positively impacts the rising prevalence of asthma. The findings from our study will inform the development of asthma management resources for pregnant women, through which the increased consumption of antioxidant-rich foods can be included in management protocols. Dietary modifications, which importantly, are in line with current national guidelines, should be a relatively simple and practical approach towards managing asthma control. Therefore, whilst the study will be conducted in women with asthma, the findings will be applicable to all pregnant women.

## Trial status

Not yet recruiting.

## Abbreviations

ACQ6: asthma control questionnaire 6; AMEA: asthma management and education advice; ANCTR: Australia and New Zealand Clinical Trials Registry; BP: blood pressure; ELISA: enzyme-linked immunosorbent assay; FENO: fraction of exhaled nitric oxide; FEV1: forced expiratory volume in 1 second; FFQ: food frequency questionnaire; F + V: fruit and vegetable; ICS: inhaled corticosteroids; IL: interleukin; IUGR: intrauterine growth restriction; LBW: low birth weight; LGA: large for gestational age; LMH: Lyell McEwin Hospital; RCT: randomized controlled trial; SGA: small for gestational age; TNF: tumor necrosis factor.

## Competing interests

The authors declare that they have no competing interests.

## Authors’ contributions

JG and VC conceived the idea. JG, LW and VC contributed to the development of the protocol. JG drafted the manuscript and LW and VC provided critical revision of the manuscript. All authors read and approved the final manuscript.
